# Role of Pneumonectomy in T1–4N2M0 Non-Small Cell Lung Cancer: A Propensity Score Matching Analysis

**DOI:** 10.3389/fonc.2022.880515

**Published:** 2022-06-20

**Authors:** Suyu Wang, Qing Wang, Wanli Zhu, Juan Wei, Di Feng, Xin Lv, Meiyun Liu

**Affiliations:** ^1^ Department of Anesthesiology, Shanghai Pulmonary Hospital, Tongji University School of Medicine, Shanghai, China; ^2^ Department of Cardiothoracic Surgery, Changzheng Hospital, Naval Medical University, Shanghai, China; ^3^ Department of Thoracic Surgery, Renji Hospital, Shanghai Jiao Tong University School of Medicine, Shanghai, China; ^4^ Department of General Surgery, Shanghai Pulmonary Hospital, Tongji University School of Medicine, Shanghai, China

**Keywords:** non-small cell lung cancer, N2 stage, pneumonectomy, chemoradiotherapy, SEER

## Abstract

**Background:**

N2 stage disease constitutes approximately 20%–30% of all non-small cell lung cancer (NSCLC). Concurrently, surgery remains the first-choice treatment for patients with N2 NSCLC if feasible. However, the role of pneumonectomy in N2 NSCLC has rarely been investigated and remains controversial.

**Methods:**

We enrolled 26,798 patients with T1–4N2M0 NSCLC (stage IIIA/IIIB) from the Surveillance, Epidemiology, and End Results (SEER) database between 2004 and 2015. We compared the overall survival (OS) and cancer-specific survival (CSS) between patients who received pneumonectomy and those who did not receive surgery. The Kaplan–Meier method, Cox regression analyses, and propensity score matching (PSM) were applied to demonstrate the effect of pneumonectomy.

**Results:**

Patients receiving pneumonectomy had a significantly better OS and CSS than those without pneumonectomy both before [adjusted-HR (95% CI): 0.461 (0.425–0.501) for OS, 0.444 (0.406–0.485) for CSS] and after PSM [adjusted-HR (95% CI): 0.499 (0.445–0.560) for OS, 0.457 (0.405–0.517) for CSS] with all *p*-values <0.001. Subgroup analysis demonstrated concordant results stratified by demographic or clinicopathological variables. In sensitivity analysis, no significant difference was observed between patients receiving single pneumonectomy and chemoradiotherapy without surgery in OS and CSS both before [unadjusted-HR (95% CI): 1.016 (0.878–1.176) for OS, 0.934 (0.794–1.099) for CSS, *p* = 0.832] and after PSM [unadjusted-HR (95% CI): 0.988 (0.799–1.222) for OS, 0.938 (0.744–1.182) for CSS] with all *p*-values >0.4.

**Conclusion:**

For patients with T1–4N2M0 NSCLC (stage IIIA/IIIB), pneumonectomy is an independent protective factor of OS and should be considered when applicable.

## Introduction

Although annual mortality has steadily declined for years, lung cancer remains one of the most common and deadly malignancies (1). As the major subtype accounting for about 85% of lung cancer, non-small cell lung cancer (NSCLC) has been subjected to multidisciplinary treatments with rapid advancements in targeted therapy, immunotherapy, and radiotherapy (2). However, surgery remains the first-line treatment for stage IA–IIIB NSCLC patients if applicable (3). According to the eighth edition of the American Joint Committee on Cancer (AJCC) TNM staging system, the N2 stage represents metastasis to lymph nodes located in the ipsilateral mediastina and/or below carina. For patients with N2 NSCLC without distant metastasis (T1–4N2M0, stage IIIA–IIIB), the treatment and prognosis vary significantly, and the heterogeneity arises from the individual physical condition, histological type, and treatment strategy (4).

Pneumonectomy is performed in patients with central giant masses or tumors involving the main bronchus or large blood vessels, particularly for N2 NSCLC patients. There are many controversies over pneumonectomy for N2 patients (5). Pneumonectomy is associated with increased in-hospital death and decreased life quality because of massive trauma and lost pulmonary function. However, pneumonectomy, which indeed completes the R0 resection of the primary malignancy, significantly decreases the tumor burden. There are limited studies and few consensuses regarding the management of N2 patients and the role of pneumonectomy (5–9). Many candidates for pneumonectomy are excluded from surgery because of the concerns regarding complications and risks. The aim of the present study was to analyze the role of pneumonectomy in N2 patients and compare the long-term outcome of N2 NSCLC with and without pneumonectomy using data from the Surveillance, Epidemiology, and End Results (SEER) database.

## Methods

### Patient Selection and Variable Extraction

We collected data from the SEER database, which included 18 cancer registries of the National Cancer Institute, using the SEER*Stat software (version 8.3.9; https://seer.cancer.gov/resources/). Patients diagnosed with T1–4N2M0 NSCLC using the American Joint Committee on Cancer (AJCC) eighth TNM classification from 2004 to 2015 were enrolled in this research. The following variables were downloaded: “Year of diagnosis”, “Age”, “Race recode”, “Sex”, “Marital status”, “Laterality”, “Primary Site—labeled”, “Histologic Type ICD-O-3”, “Grade”, “RX Summ–Surg Prim Site”, “Derived AJCC T, 6th ed (2004–2015)”, “Derived AJCC N, 6th ed (2004–2015)”, “Derived AJCC T, 7th ed (2010–2015)”, “Derived AJCC N, 7th ed (2010–2015)”, “Survival months”, “Vital status recode”, “SEER cause-specific death classification”, “Regional nodes positive”, “Regional nodes examined”, and “Sequence number of tumor”. The AJCC TNM classification was transformed into the eighth version. The exclusion criteria were as follows: (a) patients who have more than one malignant tumor; (b) patients under the age of 18 years old; (c) patients treated with surgery other than pneumonectomy; (d) patients who received a diagnosis of NSCLC based on autopsy/death certificate merely or were diagnosed without being pathologically confirmed; and (e) patients with unknown data of variables needed by our research. **Table S1** shows the selection procedure of the study cohort. Overall survival (OS) defined as time from diagnosis to all-cause death was the primary endpoint, and cancer-specific survival (CSS) defined as time from diagnosis to NSCLC death was the second endpoint. The latest follow-up time was December 31, 2016.

### Ethical Statement

As in other SEER-based studies, no personally identifying patient information was included in the SEER data, and the request for approval of the institutional review committee and consent of the patients were waived in this study. We conducted the research in accordance with the Declaration of Helsinki (as revised in 2013).

### Statistical Analysis

Two statistical software were used in analyses: EmpowerStats (version 2.0; http://www.empowerstats.com) and R software (version 4.0.4; http://www.r-project.org). *p*-value <0.05 was regarded as statistically significant. Categorical variables were presented as number (proportion). Chi-square test or Fisher’s precision probability test was performed to compare categorical variables, as appropriate.

Utilizing the X-tile software from https://medicine.yale.edu/lab/rimm/research/software/, continuous variables, including age and regional nodes examined/positive, were trichotomized to achieve the largest difference in OS between subgroups (10, 11). We divided the enrolled patients into two groups, namely, patients receiving pneumonectomy and no surgery. Propensity score matching (PSM) was used to balance the baseline characteristics, with a caliper of 0.02. All baseline characteristics were included in the PSM logistic model except for regional nodes examined, and regional nodes positive for these two variables were highly determined by whether surgery was performed. These two variables were analyzed in the surgery group alone to determine their influence on OS and CSS (11). The OS and CSS of the two groups were compared before and after PSM with a Kaplan–Meier survival curve and the log-rank test. Univariable Cox regression analysis was conducted for all variables, and those potentially influenced OS and CSS with a *p*-value <0.1 were subjected to multivariable analysis. A subgroup analysis of different groups was also completed to compare no surgery and pneumonectomy. In addition, we conducted the sensitivity analysis by comparing the OS and CSS between the patients receiving chemoradiotherapy and single pneumonectomy using the Cox regression analysis performed.

## Results

### Baseline Characteristics

As shown in **Table 1**, we enrolled a total of 26,798 T1–4N2M0 NSCLC patients diagnosed between 2004 and 2015 in the SEER database. According to treatment strategy, we divided them into the no-surgery group (*n* = 25,933) and the pneumonectomy group (*n* = 865). Before PSM, there was a significant difference between the two groups in all included variables, namely, year of diagnosis, age, gender, race, marital status, laterality, primary site, histologic type, differentiation, T, regional nodes examined, regional nodes positive, and radiotherapy or chemotherapy (all *p* < 0.005). However, we conducted the PSM to match paired patients and decrease differences in baseline characteristics. After PSM, a total of 782 pairs of patients were included, with no statistical difference in variables used for PSM (all *p* > 0.05).

**Table 1:** Baseline characteristics of stage T1–4N2M0 NSCLC patients.

Variables	Before PSM			After PSM		
	No-surgery (*n* = 25,933)	Pneumonectomy (*n* = 865)	*p*	No-surgery (*n* = 782)	Pneumonectomy (*n* = 782)	*p*
**Year of diagnosis**			<0.001			0.507
2004–2009	12,056 (46.5%)	509 (58.8%)		436 (55.8%)	449 (57.4%)	
2010–2015	13,877 (53.5%)	356 (41.2%)		346 (44.2%)	333 (42.6%)	
**Age**			<0.001			0.767
<64 years old	8,409 (32.4%)	554 (64.0%)		483 (61.8%)	483 (61.8%)	
64–76 years old	11,156 (43.0%)	269 (31.1%)		263 (33.6%)	257 (32.9%)	
>76 years old	6,368 (24.6%)	42 (4.9%)		36 (4.6%)	42 (5.4%)	
**Gender**			<0.001			0.123
Male	14,791 (57.0%)	552 (63.8%)		528 (67.5%)	499 (63.8%)	
Female	11,142 (43.0%)	313 (36.2%)		254 (32.5%)	283 (36.2%)	
**Race**			<0.001			0.099
White	20,695 (79.8%)	709 (82.0%)		670 (85.7%)	639 (81.7%)	
Black	3,710 (14.3%)	89 (10.3%)		69 (8.8%)	85 (10.9%)	
Other	1,528 (5.9%)	67 (7.7%)		43 (5.5%)	58 (7.4%)	
**Marital status**			<0.001			0.615
Single	3,625 (14.0%)	111 (12.8%)		97 (12.4%)	100 (12.8%)	
Married	12,833 (49.5%)	531 (61.4%)		486 (62.1%)	472 (60.4%)	
Separated/Divorced/Widowed	8,476 (32.7%)	196 (22.7%)		182 (23.3%)	185 (23.7%)	
Unknown	999 (3.9%)	27 (3.1%)		17 (2.2%)	25 (3.2%)	
**Laterality**			<0.001			0.51
Right	16,607 (64.0%)	370 (42.8%)		368 (47.1%)	355 (45.4%)	
Left	9,326 (36.0%)	495 (57.2%)		414 (52.9%)	427 (54.6%)	
**Primary site**			<0.001			0.726
Main bronchus	1,630 (6.3%)	79 (9.1%)		63 (8.1%)	69 (8.8%)	
Upper lobe	15,627 (60.3%)	461 (53.3%)		463 (59.2%)	436 (55.8%)	
Middle lobe	1,030 (4.0%)	36 (4.2%)		37 (4.7%)	35 (4.5%)	
Lower lobe	6,114 (23.6%)	195 (22.5%)		169 (21.6%)	180 (23.0%)	
Overlapping lesion of lung	272 (1.0%)	69 (8.0%)		31 (4.0%)	37 (4.7%)	
Unknown	1,260 (4.9%)	25 (2.9%)		19 (2.4%)	25 (3.2%)	
**Histologic type**			0.002			0.759
Adenocarcinoma	8,723 (33.6%)	314 (36.3%)		278 (35.5%)	290 (37.1%)	
Squamous cell	10,820 (41.7%)	384 (44.4%)		352 (45.0%)	338 (43.2%)	
Other	6,390 (24.6%)	167 (19.3%)		152 (19.4%)	154 (19.7%)	
**Differentiation**			<0.001			0.735
Grade I	642 (2.5%)	31 (3.6%)		19 (2.4%)	28 (3.6%)	
Grade II	4,021 (15.5%)	272 (31.4%)		235 (30.1%)	231 (29.5%)	
Grade III	8,231 (31.7%)	421 (48.7%)		398 (50.9%)	388 (49.6%)	
Grade IV	465 (1.8%)	32 (3.7%)		26 (3.3%)	26 (3.3%)	
Unknown	12,574 (48.5%)	109 (12.6%)		104 (13.3%)	109 (13.9%)	
**T**			<0.001			0.053
T1	3,567 (13.8%)	48 (5.5%)		29 (3.7%)	48 (6.1%)	
T2	6,752 (26.0%)	295 (34.1%)		291 (37.2%)	254 (32.5%)	
T3	6,033 (23.3%)	224 (25.9%)		184 (23.5%)	197 (25.2%)	
T4	9,581 (36.9%)	298 (34.5%)		278 (35.5%)	283 (36.2%)	
**Regional nodes examined**			<0.001			<0.001
0–6	21,548 (83.1%)	144 (16.6%)		694 (88.7%)	133 (17.0%)	
7–23	315 (1.2%)	502 (58.0%)		5 (0.6%)	447 (57.2%)	
>23	42 (0.2%)	118 (13.6%)		1 (0.1%)	111 (14.2%)	
Unknown	4,028 (15.5%)	101 (11.7%)		82 (10.5%)	91 (11.6%)	
**Regional nodes positive**			<0.001			<0.001
0	568 (2.2%)	96 (11.1%)		13 (1.7%)	87 (11.1%)	
1–5	2,293 (8.8%)	485 (56.1%)		73 (9.3%)	434 (55.5%)	
>5	103 (0.4%)	191 (22.1%)		1 (0.1%)	174 (22.3%)	
Unknown	22,969 (88.6%)	93 (10.8%)		695 (88.9%)	87 (11.1%)	
**Radiotherapy or chemotherapy**			<0.001			0.514
No	5,585 (21.5%)	222 (25.7%)		205 (26.2%)	201 (25.7%)	
Radiotherapy	3,548 (13.7%)	25 (2.9%)		22 (2.8%)	25 (3.2%)	
Chemotherapy	2,997 (11.6%)	258 (29.8%)		177 (22.6%)	200 (25.6%)	
Both	13,803 (53.2%)	360 (41.6%)		378 (48.3%)	356 (45.5%)	
**All-cause death**	22,589 (87.1%)	630 (72.8%)	<0.001	691 (88.4%)	567 (72.5%)	<0.001
**Cancer-specific death**	20,223 (78.0%)	538 (62.2%)	<0.001	638 (81.6%)	479 (61.3%)	<0.001

Categorical variables are presented with number (percentage). NSCLC, non-small cell lung cancer; PSM, propensity score matching.

### Overall Survival

**Figure 1:**
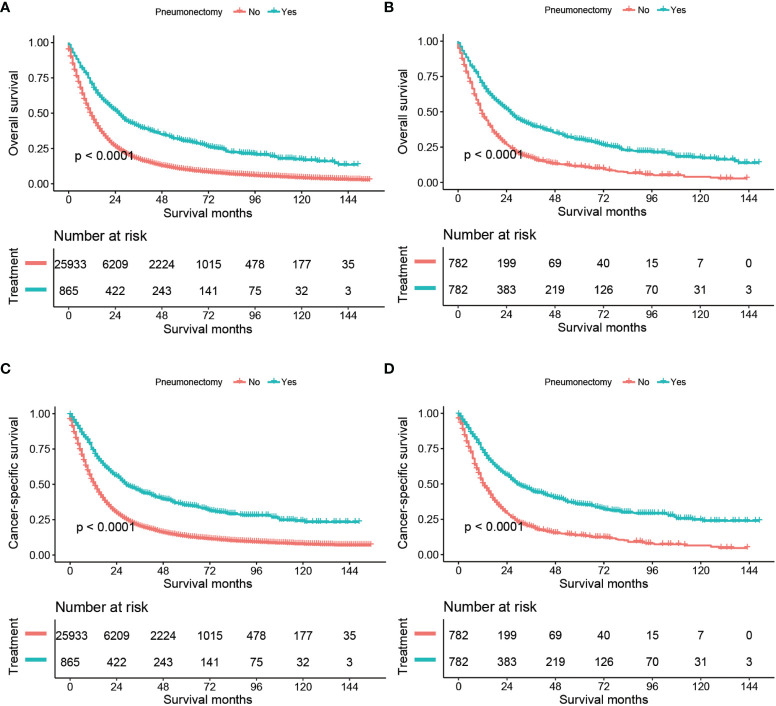
Kaplan–Meier estimates of OS and CSS for stage T1–4N2M0 NSCLC patients comparing no surgery with pneumonectomy: OS before PSM **(A)**, OS after PSM **(B)**, CSS before PSM **(C)**, and CSS after PSM **(D)**. OS, overall survival; CSS, cancer-specific survival; NSCLC, non-small cell lung cancer, PSM, propensity score matching.

We compared the OS and CSS between the no-surgery group and the pneumonectomy group, shown in **Figure 1**. The median follow-up time [interquartile range (IQR)] was 79 (45–117) months. Before PSM, the median OS of the no-surgery and pneumonectomy group was 11 months and 25 months, and the median CSS of these two groups was 12 and 29, respectively. There were 22,589 (87.1%) deaths in the no-surgery group and 630 (72.8%) all-cause deaths in the pneumonectomy group during the follow-up, while the cancer-specific deaths for these two groups were 20,223 (78.0%) and 538 (62.2%), respectively. The 1-year, 3-year, and 5-year OS rates [95% confidence interval (95% CI)] of the no-surgery group were 47.34% (46.73%–47.95%), 17.17% (16.70%–17.67%), and 9.89% (9.48%–10.32%), respectively, while the 1-year, 3-year, and 5-year OS rates of the pneumonectomy group were 70.15% (67.16%–73.27%), 40.55% (37.32%–44.07%), and 30.22% (27.12%–33.66%), respectively. The 1-year, 3-year, and 5-year CSS rates (95% CI) of the no-surgery group were 50.74% (50.12%–51.37%), 20.26% (19.72%–20.81%), and 12.91% (12.42%–13.42%), respectively, while the 1-year, 3-year, and 5-year CSS rates of the pneumonectomy group were 73.65% (70.72%–76.70%), 45.01% (41.62%–48.67%), and 34.98% (31.63%–38.70%), respectively. The log-rank test showed that the pneumonectomy group had a significantly higher OS and CSS than the no-surgery group before and after PSM (*p* < 0.0001; **Figure 1**).

### Univariable and Multivariable Analyses

We conducted the univariable and multivariable analyses to screen the risk factors for OS and CSS of T1–4N2M0 NSCLC patients (**Tables 2** and **3**). Before PSM, the univariable analysis revealed that patients who received pneumonectomy, who were diagnosed in 2010–2015, who were younger, who were female, with a non-white race, with a marital status other than separated/divorced/widowed, with left lung disease, with upper lobe disease, with adenocarcinoma, with a higher differentiated degree, with a lower T classification, and who received chemotherapy or chemoradiotherapy achieved better OS or CSS outcome (*p* < 0.05). Multivariable analysis demonstrated that surgery, year of diagnosis, age, gender, race, marital status, primary site, histological type, differentiation, T classification, and radiotherapy or chemotherapy were independent risk factors for OS of T1–4N2M0 NSCLC patients; the same results were found for CSS except that laterality was also independently related to CSS. The unadjusted-HR for pneumonectomy versus no surgery before [unadjusted-HR (95% CI): 0.523 (0.483–0.566) for OS, 0.509 (0.467–0.555) for CSS] and after PSM [unadjusted-HR (95% CI): 0.525 (0.469–0.588) for OS, 0.487 (0.432–0.549) for CSS] with all *p*-values <0.001 and adjusted-HR for pneumonectomy versus no surgery before [adjusted-HR (95% CI): 0.461 (0.425–0.501) for OS, 0.444 (0.406–0.485) for CSS] and after PSM [adjusted-HR (95% CI): 0.499 (0.445–0.560) for OS, 0.457 (0.405–0.517) for CSS] with all *p*-values <0.001 showed better survival outcome of patients who underwent pneumonectomy.

**Table 2:** Cox regression analysis of the influence of pneumonectomy on OS in stage T1–4N2M0 NSCLC patients.

Variables	Before PSM				After PSM			
	Univariable analysis		Multivariable analysis		Univariable analysis		Multivariable analysis	
	HR (95% CI)	*p*	HR (95% CI)	*p*	HR (95% CI)	*p*	HR (95% CI)	*p*
**Surgery**								
No	1		1		1		1	
Pneumonectomy	0.523 (0.483–0.566)	<0.001	0.461 (0.425–0.501)	<0.001	0.525 (0.469–0.588)	<0.001	0.499 (0.445–0.560)	<0.001
**Year of diagnosis**								
2004–2009	1		1		1		1	
2010–2015	0.911 (0.887–0.935)	<0.001	0.922 (0.898–0.948)	<0.001	0.875 (0.779–0.983)	0.025	0.891 (0.791–1.002)	0.055
**Age**								
<64 years old	1		1		1		1	
64–76 years old	1.247 (1.210–1.285)	<0.001	1.177 (1.141–1.214)	<0.001	1.285 (1.142–1.447)	<0.001	1.182 (1.046–1.335)	0.007
>76 years old	1.634 (1.578–1.691)	<0.001	1.303 (1.255–1.353)	<0.001	1.950 (1.530–2.485)	<0.001	1.660 (1.292–2.132)	<0.001
**Gender**								
Male	1		1		1		1	
Female	0.872 (0.850–0.896)	<0.001	0.849 (0.826–0.873)	<0.001	0.853 (0.758–0.959)	0.008	0.924 (0.820–1.040)	0.19
**Race**								
White	1		1		1		1	
Black	0.917 (0.883–0.952)	<0.001	0.925 (0.891–0.961)	<0.001	0.955 (0.792–1.151)	0.628	1.002 (0.829–1.211)	0.987
Other	0.849 (0.802–0.898)	<0.001	0.832 (0.786–0.880)	<0.001	0.790 (0.622–1.003)	0.053	0.813 (0.639–1.034)	0.092
**Marital status**								
Single	1		1		1			
Married	0.957 (0.920–0.995)	0.028	0.931 (0.894–0.970)	<0.001	0.878 (0.739–1.042)	0.137		
Separated/Divorced/Widowed	1.103 (1.058–1.150)	<0.001	1.023 (0.980–1.068)	0.306	1.103 (0.910–1.337)	0.316		
Unknown	1.035 (0.960–1.116)	0.373	0.973 (0.902–1.050)	0.487	1.034 (0.713–1.498)	0.861		
**Laterality**								
Right	1		1		1			
Left	0.968 (0.942–0.994)	0.016	0.977 (0.951–1.004)	0.095	0.943 (0.844–1.053)	0.298		
**Primary site**								
Main bronchus	1		1		1			
Upper lobe	0.885 (0.839–0.934)	<0.001	0.869 (0.824–0.917)	<0.001	0.883 (0.721–1.081)	0.227		
Middle lobe	0.934 (0.861–1.014)	0.102	0.947 (0.872–1.029)	0.201	0.977 (0.711–1.342)	0.886		
Lower lobe	0.994 (0.939–1.052)	0.832	0.950 (0.896–1.006)	0.08	0.992 (0.795–1.237)	0.942		
Overlapping lesion of lung	0.986 (0.871–1.116)	0.822	1.016 (0.897–1.152)	0.798	0.912 (0.655–1.269)	0.584		
Unknown	1.130 (1.046–1.221)	0.002	1.028 (0.951–1.111)	0.489	1.203 (0.825–1.755)	0.337		
**Histologic type**								
Adenocarcinoma	1		1		1			
Squamous cell	1.264 (1.227–1.303)	<0.001	1.167 (1.131–1.204)	<0.001	1.038 (0.917–1.175)	0.556		
Other	1.233 (1.192–1.276)	<0.001	1.144 (1.104–1.185)	<0.001	0.979 (0.838–1.144)	0.79		
**Differentiation**								
Grade I	1		1		1			
Grade II	1.131 (1.035–1.236)	0.007	1.171 (1.071–1.281)	<0.001	1.194 (0.836–1.705)	0.329		
Grade III	1.179 (1.082–1.284)	<0.001	1.236 (1.133–1.348)	<0.001	1.280 (0.902–1.817)	0.167		
Grade IV	1.338 (1.182–1.514)	<0.001	1.391 (1.227–1.578)	<0.001	1.443 (0.921–2.262)	0.109		
Unknown	1.124 (1.032–1.223)	0.007	1.155 (1.060–1.258)	<0.001	1.167 (0.804–1.693)	0.417		
**T**								
T1	1		1		1		1	
T2	1.211 (1.159–1.265)	<0.001	1.238 (1.184–1.294)	<0.001	1.301 (0.992–1.708)	0.057	1.308 (0.995–1.719)	0.054
T3	1.333 (1.274–1.394)	<0.001	1.398 (1.336–1.464)	<0.001	1.356 (1.026–1.793)	0.033	1.543 (1.163–2.046)	0.003
T4	1.469 (1.408–1.531)	<0.001	1.584 (1.518–1.654)	<0.001	1.401 (1.067–1.838)	0.015	1.647 (1.250–2.171)	<0.001
**Radiotherapy or chemotherapy**								
No	1		1		1		1	
Radiotherapy	0.704 (0.674–0.734)	<0.001	0.647 (0.620–0.676)	<0.001	0.710 (0.512–0.985)	0.041	0.729 (0.525–1.012)	0.059
Chemotherapy	0.509 (0.487–0.533)	<0.001	0.530 (0.506–0.555)	<0.001	0.584 (0.500–0.682)	<0.001	0.587 (0.501–0.687)	<0.001
Both	0.386 (0.374–0.399)	<0.001	0.378 (0.365–0.391)	<0.001	0.531 (0.465–0.607)	<0.001	0.497 (0.433–0.572)	<0.001

OS, overall survival; NSCLC, non-small cell lung cancer; PSM, propensity score matching; HR, hazard ratio; CI, confidence interval.

**Table 3:** Cox regression analysis of the influence of pneumonectomy on CSS in stage T1–4N2M0 NSCLC patients.

Variables	Before PSM				After PSM			
	Univariable analysis		Multivariable analysis		Univariable analysis		Multivariable analysis	
	HR (95% CI)	*p*	HR (95% CI)	*p*	HR (95% CI)	*p*	HR (95% CI)	*p*
**Surgery**								
No	1		1		1		1	
Pneumonectomy	0.509 (0.467–0.555)	<0.001	0.444 (0.406–0.485)	<0.001	0.487 (0.432–0.549)	<0.001	0.457 (0.405–0.517)	<0.001
**Year of diagnosis**								
2004–2009	1		1		1		1	
2010–2015	0.908 (0.883–0.933)	<0.001	0.917 (0.891–0.943)	<0.001	0.887 (0.785–1.003)	0.055	0.888 (0.784–1.007)	0.064
**Age**								
<64 years old	1		1		1		1	
64–76 years old	1.194 (1.156–1.232)	<0.001	1.129 (1.092–1.166)	<0.001	1.185 (1.044–1.345)	0.009	1.098 (0.963–1.251)	0.163
>76 years old	1.555 (1.500–1.613)	<0.001	1.250 (1.201–1.300)	<0.001	1.755 (1.348–2.286)	<0.001	1.558 (1.185–2.048)	0.002
**Gender**								
Male	1		1		1			
Female	0.879 (0.855–0.904)	<0.001	0.861 (0.836–0.887)	<0.001	0.911 (0.805–1.030)	0.137		
**Race**								
White	1		1		1		1	
Black	0.911 (0.875–0.947)	<0.001	0.912 (0.876–0.950)	<0.001	0.938 (0.768–1.146)	0.532	0.925 (0.752–1.138)	0.462
Other	0.873 (0.823–0.926)	<0.001	0.854 (0.805–0.906)	<0.001	0.784 (0.609–1.010)	0.06	0.804 (0.623–1.038)	0.094
**Marital status**								
Single	1		1		1		1	
Married	0.962 (0.923–1.003)	0.071	0.943 (0.903–0.984)	0.007	0.847 (0.708–1.014)	0.071	0.785 (0.650–0.946)	0.011
Separated/Divorced/Widowed	1.093 (1.047–1.142)	<0.001	1.025 (0.979–1.072)	0.293	1.052 (0.859–1.287)	0.626	0.943 (0.766–1.162)	0.583
Unknown	1.033 (0.954–1.119)	0.418	0.979 (0.903–1.060)	0.599	1.089 (0.746–1.589)	0.66	1.024 (0.699–1.499)	0.904
**Laterality**								
Right	1		1		1			
Left	0.958 (0.931–0.986)	0.003	0.969 (0.942–0.998)	0.034	0.907 (0.806–1.020)	0.103		
**Primary site**								
Main bronchus	1		1		1			
Upper lobe	0.867 (0.820–0.917)	<0.001	0.857 (0.810–0.907)	<0.001	0.901 (0.726–1.117)	0.342		
Middle lobe	0.913 (0.838–0.996)	0.04	0.933 (0.854–1.018)	0.118	0.938 (0.666–1.321)	0.715		
Lower lobe	0.971 (0.914–1.031)	0.33	0.938 (0.883–0.997)	0.04	0.973 (0.769–1.232)	0.822		
Overlapping lesion of lung	0.966 (0.847–1.102)	0.603	0.991 (0.868–1.132)	0.894	0.894 (0.627–1.274)	0.535		
Unknown	1.114 (1.027–1.209)	0.009	1.009 (0.930–1.095)	0.827	1.193 (0.799–1.782)	0.388		
**Histologic type**								
Adenocarcinoma	1		1		1			
Squamous cell	1.235 (1.196–1.275)	<0.001	1.136 (1.099–1.174)	<0.001	0.959 (0.842–1.094)	0.536		
Other	1.226 (1.182–1.271)	<0.001	1.130 (1.088–1.174)	<0.001	0.963 (0.818–1.134)	0.654		
**Differentiation**								
Grade I	1		1		1			
Grade II	1.158 (1.053–1.274)	0.002	1.200 (1.091–1.321)	<0.001	1.105 (0.769–1.589)	0.589		
Grade III	1.226 (1.118–1.344)	<0.001	1.277 (1.164–1.402)	<0.001	1.175 (0.823–1.679)	0.374		
Grade IV	1.370 (1.200–1.564)	<0.001	1.415 (1.237–1.619)	<0.001	1.259 (0.787–2.016)	0.336		
Unknown	1.166 (1.064–1.277)	<0.001	1.196 (1.091–1.311)	<0.001	1.068 (0.729–1.564)	0.736		
**T**								
T1	1		1		1		1	
T2	1.266 (1.207–1.328)	<0.001	1.297 (1.236–1.360)	<0.001	1.509 (1.109–2.054)	0.009	1.509 (1.106–2.057)	0.009
T3	1.430 (1.363–1.501)	<0.001	1.505 (1.433–1.580)	<0.001	1.546 (1.127–2.121)	0.007	1.756 (1.276–2.417)	<0.001
T4	1.608 (1.537–1.682)	<0.001	1.734 (1.656–1.816)	<0.001	1.644 (1.208–2.238)	0.002	1.921 (1.406–2.626)	<0.001
**Radiotherapy or chemotherapy**								
No	1		1		1		1	
Radiotherapy	0.707 (0.675–0.740)	<0.001	0.651 (0.621–0.681)	<0.001	0.713 (0.502–1.011)	0.058	0.757 (0.532–1.076)	0.121
Chemotherapy	0.535 (0.510–0.561)	<0.001	0.550 (0.525–0.578)	<0.001	0.611 (0.518–0.720)	<0.001	0.618 (0.522–0.731)	<0.001
Both	0.398 (0.384–0.412)	<0.001	0.384 (0.370–0.398)	<0.001	0.548 (0.475–0.631)	<0.001	0.507 (0.437–0.589)	<0.001

CSS, cancer-specific survival; NSCLC, non-small cell lung cancer; PSM, propensity score matching; HR, hazard ratio; CI, confidence interval.

To analyze the influence of regional nodes examined/positive on OS and CSS of patients receiving pneumonectomy, we also conducted univariable and multivariable analyses (**Table 4**). The results demonstrated that the regional nodes examined was not significantly related to OS, while the regional nodes positive was an independent risk factor for OS and CSS. The adjusted-HR (95% CI) of regional nodes positive 1–5 compared with 0 was 1.287 (0.962–1.721) for OS with *p* = 0.089 and 1.464 (1.055–2.031) for CSS with *p* = 0.022. The adjusted-HR (95% CI) of regional nodes positive >5 compared with 0 was 1.874 (1.371–2.562) for OS and 2.211 (1.562–3.130) for CSS with all *p*-values <0.001.

**Table 4:** Cox regression analysis of influence of regional nodes examined and regional nodes positive on OS and CSS in stage T1–4N2M0 NSCLC patients who underwent pneumonectomy.

Variables	OS				CSS			
	Univariable analysis		Multivariable analysis		Univariable analysis		Multivariable analysis	
	HR (95% CI)	*p*	HR (95% CI)	*p*	HR (95% CI)	*p*	HR (95% CI)	*p*
**Regional nodes examined**								
0–6	1				1			
7–23	1.037 (0.834–1.290)	0.745			0.957 (0.757–1.209)	0.711		
>23	1.257 (0.946–1.669)	0.115			1.272 (0.943–1.716)	0.115		
Unknown	0.988 (0.734–1.329)	0.935			0.995 (0.726–1.363)	0.974		
**Regional nodes positive**								
0	1		1		1		1	
1–5	1.383 (1.046–1.829)	0.023	1.287 (0.962–1.721)	0.089	1.503 (1.093–2.066)	0.012	1.464 (1.055–2.031)	0.022
>5	1.994 (1.475–2.697)	<0.001	1.874 (1.371–2.562)	<0.001	2.280 (1.624–3.201)	<0.001	2.211 (1.562–3.130)	<0.001
Unknown	1.589 (1.124–2.247)	0.009	1.514 (1.066–2.150)	0.021	1.891 (1.291–2.772)	0.001	1.845 (1.257–2.707)	0.002
**Year of diagnosis**								
2004–2009	1		1		1		1	
2010–2015	0.806 (0.680–0.955)	0.013	0.805 (0.676–0.958)	0.015	0.803 (0.669–0.963)	0.018	0.771 (0.641–0.927)	0.006
**Age**								
<64 years old	1		1		1		1	
64–76 years old	1.259 (1.063–1.492)	0.008	1.118 (0.936–1.337)	0.219	1.077 (0.892–1.299)	0.44	0.985 (0.811–1.197)	0.88
>76 years old	2.350 (1.675–3.298)	<0.001	2.131 (1.501–3.025)	<0.001	1.997 (1.366–2.920)	<0.001	1.822 (1.234–2.690)	0.003
**Gender**								
Male	1		1		1		1	
Female	0.787 (0.667–0.928)	0.004	0.759 (0.642–0.897)	0.001	0.853 (0.715–1.017)	0.076	0.816 (0.683–0.975)	0.025
**Race**								
White	1				1			
Black	0.920 (0.704–1.202)	0.542			0.872 (0.649–1.172)	0.364		
Other	1.002 (0.747–1.343)	0.99			1.013 (0.740–1.385)	0.937		
**Marital status**								
Single	1		1		1			
Married	1.015 (0.793–1.301)	0.903	0.951 (0.738–1.226)	0.7	0.935 (0.720–1.212)	0.61		
Separated/Divorced/Widowed	1.280 (0.971–1.687)	0.08	1.161 (0.873–1.544)	0.305	1.151 (0.859–1.543)	0.346		
Unknown	1.399 (0.869–2.251)	0.167	1.547 (0.954–2.507)	0.077	1.427 (0.875–2.327)	0.154		
**Laterality**								
Right	1		1		1		1	
Left	0.869 (0.743–1.018)	0.082	0.814 (0.693–0.956)	0.012	0.806 (0.680–0.955)	0.013	0.778 (0.654–0.925)	0.004
**Primary site**								
Main bronchus	1				1			
Upper lobe	1.030 (0.769–1.380)	0.843			1.038 (0.756–1.426)	0.817		
Middle lobe	1.096 (0.684–1.755)	0.703			1.099 (0.659–1.833)	0.718		
Lower lobe	1.272 (0.929–1.742)	0.133			1.222 (0.867–1.721)	0.252		
Overlapping lesion of lung	1.090 (0.739–1.608)	0.664			1.143 (0.754–1.732)	0.529		
Unknown	1.291 (0.755–2.209)	0.351			1.227 (0.683–2.207)	0.494		
**Histologic type**								
Adenocarcinoma	1				1			
Squamous cell	0.977 (0.821–1.164)	0.798			0.888 (0.736–1.072)	0.217		
Other	0.918 (0.736–1.145)	0.447			0.884 (0.697–1.120)	0.306		
**Differentiation**								
Grade I	1		1		1			
Grade II	1.259 (0.777–2.041)	0.35	1.326 (0.811–2.167)	0.261	1.139 (0.692–1.876)	0.609		
Grade III	1.379 (0.857–2.218)	0.185	1.435 (0.886–2.326)	0.142	1.237 (0.758–2.021)	0.395		
Grade IV	1.872 (1.031–3.400)	0.04	2.157 (1.175–3.961)	0.013	1.670 (0.892–3.127)	0.109		
Unknown	1.205 (0.720–2.016)	0.477	1.429 (0.840–2.430)	0.188	1.091 (0.639–1.862)	0.75		
**T**								
T1	1				1		1	
T2	1.132 (0.792–1.617)	0.497			1.240 (0.828–1.857)	0.297	1.283 (0.854–1.927)	0.231
T3	1.260 (0.874–1.816)	0.216			1.384 (0.916–2.089)	0.123	1.494 (0.985–2.265)	0.059
T4	1.284 (0.897–1.836)	0.172			1.421 (0.948–2.130)	0.089	1.601 (1.062–2.415)	0.025
**Radiotherapy or chemotherapy**								
No	1		1		1		1	
Radiotherapy	0.672 (0.419–1.078)	0.099	0.638 (0.396–1.029)	0.065	0.599 (0.346–1.037)	0.067	0.562 (0.324–0.975)	0.04
Chemotherapy	0.572 (0.465–0.704)	<0.001	0.620 (0.500–0.768)	<0.001	0.640 (0.512–0.801)	<0.001	0.653 (0.520–0.821)	<0.001
Both	0.591 (0.488–0.715)	<0.001	0.634 (0.517–0.777)	<0.001	0.619 (0.502–0.763)	<0.001	0.623 (0.500–0.777)	<0.001

OS, overall survival; CSS, cancer-specific survival; NSCLC, non-small cell lung cancer; HR, hazard ratio; CI, confidence interval.

### Subgroup and Sensitivity Analysis

**Figure 2:**
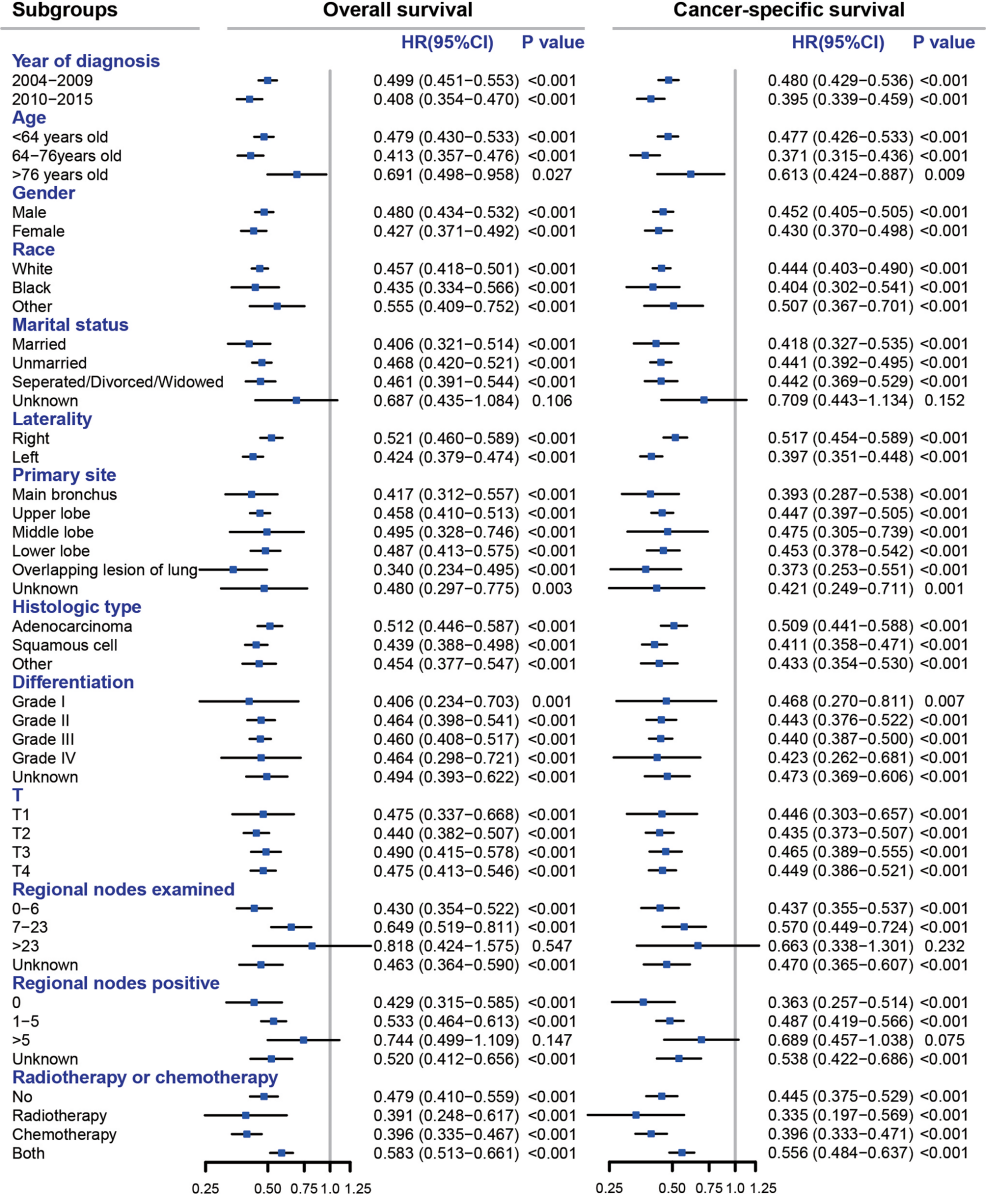
Subgroup analyses comparing pneumonectomy with no surgery for stage T1–4N2M0 NSCLC patients. All HRs were calculated by adjusting year of diagnosis, age, gender, race, marital status, laterality, primary site, histologic type, differentiation, T, and radiotherapy or chemotherapy except for the subgroup variable itself. NSCLC, non-small cell lung cancer; HR, hazard ratio; CI, confidence interval.

We compared the OS and CSS of the no-surgery group and the pneumonectomy group in subgroups of different variables (**Figure 2**). The pneumonectomy cohort demonstrated significantly higher OS and CSS rates than the no-surgery cohort in all subgroups, including the year of diagnosis, age, gender, race, marital status, laterality, histological type, primary site, differentiation, T classification, and radiotherapy or chemotherapy (all HRs < 1 with most *p*-values < 0.05). Only the HR for unknown marital status, >23 regional nodes examined, and >5 regional nodes positive group was not statistically significant.

To further validate the role of pneumonectomy in treating patients with T1–4N2M0 NSCLC, we conducted a sensitivity analysis by comparing the OS and CSS between patients who underwent chemoradiotherapy without surgery and those who received single pneumonectomy. **Table S2** shows the baseline characteristics of T1–4N2M0 NSCLC patients who underwent chemoradiotherapy (*n* = 13,803) or single pneumonectomy (*n* = 222). The survival analysis demonstrated no significant disparity between chemoradiotherapy and single pneumonectomy in OS and CSS both before and after PSM (all *p*-values >0.4), as shown in **Figure S1**. In the univariable Cox regression analysis, the unadjusted-HR (95% CI) of single pneumonectomy vs. chemoradiotherapy without surgery for OS was 1.016 (0.878–1.176) with *p* = 0.832 before PSM and 0.988 (0.799–1.222) with *p* = 0.913 after PSM (**Table S3**). Similarly, the unadjusted-HR (95% CI) for CSS was 0.934 (0.794–1.099) with *p* = 0.413 before PSM and 0.938 (0.744–1.182) with *p* = 0.586 after PSM (**Table S4**).

## Discussion

Pneumonectomy requires resection of a unilateral lung, leading to an apparent loss of pulmonary function, a high incidence of postoperative complications, and a significant in-hospital mortality (12). A prospective multicenter randomized trial reported a pneumonectomy mortality of 26% after induction chemoradiation in NSCLC (6). A meta-analysis summarized 27 studies from 1990 to 2010 describing pneumonectomy after neoadjuvant therapy, indicating that 30-day and 90-day perioperative mortalities were 7% and 12% overall (13). Therefore, pneumonectomy is gradually considered cautiously for locally advanced NSCLC, with a significant decline in the use of pneumonectomy over the past two decades in the National Cancer Database (14). This decline might also be caused by the development of new drugs of target therapy, immunotherapy, and new technologies pertaining to radiotherapy that could be applied in neoadjuvant therapy and downstage the disease, making some indications for pneumonectomy disappear and less aggressive surgeries performed instead. However, the role of pneumonectomy in NSCLC, especially its long-term outcomes, has minimal evidence. In this study, we enrolled all T1–4N2M0 NSCLC patients receiving pneumonectomy or no surgery from 2004 to 2015 in the SEER database that includes nearly 28% of the US population (15). We found that patients receiving pneumonectomy had significantly better long-term survival compared with patients who had no surgery.

According to the eighth AJCC TNM staging system, the N2 stage indicates ipsilateral and/or subcarinal mediastinal lymph node metastasis. Patients with N2 stage and without distant metastasis are classified as stage III (IIIA, T1–2N2M0; IIIB, T3-4N2M0) (16). The treatment and prognosis of N2 NSCLC patients are heterogeneous, and the role of surgical resection remains controversial. Wang et al. analyzed 4,267 N2 patients in the SEER database and developed a nomogram for prognosis prediction. They demonstrated that a multidisciplinary team should decide whether or not to perform surgical resection in N2 patients (17). For N2 patients with a central giant mass or tumor involving the main bronchus or large blood vessels, pneumonectomy is the primary surgical technique for R0 resection, usually accompanied by neoadjuvant chemoradiotherapy or induction therapy (5, 18). A study analyzed 83,913 N2 patients (T1–3, N2, M0, cStage IIIA) in the National Cancer Database between 1999 and 2011 and assessed the 5-year survival from highest to lowest depending on the treatment: patients treated with surgery in combination with chemotherapy, radiation, or both (38%), followed by surgery alone (30%), nonsurgical treatment (11%), and worst for untreated patients (5%) (14). The present study also found that compared with no surgery, pneumonectomy was associated with higher OS and CSS rates. Pneumonectomy decreased nearly half the death risks. The subgroup analysis demonstrated that the pneumonectomy was a protective factor in all subgroups with all point estimate HRs <1 with most *p*-values <0.05, and only three subgroups—unknown marital status, >23 regional nodes examined, and >5 regional nodes positive group—achieved *p*-values >0.05. We proposed that the reason was the small sample size of these subgroups, leading to a low power.

Similar to a previous study (17), we conducted univariable and multivariable analysis and found that N2 patients who received pneumonectomy, who were diagnosed in 2010–2015, who were younger, who were female, with a non-white race, with a marital status other than separated/divorced/widowed, with upper lobe disease, with adenocarcinoma, with a higher differentiated degree, with a lower T classification, and who received chemotherapy or/and radiotherapy were significantly associated with better long-term outcome. Chemotherapy and radiotherapy have been proven to be effective for NSCLC patients. However, chemotherapy or/and radiotherapy could not replace pneumonectomy. A multi-institutional study compared neoadjuvant chemoradiotherapy or chemotherapy plus surgery (CRTS) with definitive chemoradiotherapy (dCRT) in 247 T1–T3N2M0 NSCLC patients, and the surgery consisted of either lobectomy (97 patients; 82.2%) or pneumonectomy (21 patients; 17.8%). They found that CRTS yields better OS and PFS than dCRT (19). To further validate our findings, we conducted the sensitivity analysis by comparing chemoradiotherapy with single pneumonectomy, which demonstrated that there was no significant difference in long-term outcomes of patients receiving chemoradiotherapy and single pneumonectomy. The result proved that pneumonectomy could achieve a non-inferior outcome than chemoradiotherapy.

Nevertheless, pneumonectomy should be applied after comprehensive preoperative evaluation with strict surgical indications. Only 865 patients received pneumonectomy in this study, accounting for 3.2% of the entire study cohort. A study summarized the application of pneumonectomy for primary lung cancer in the Netherlands, and they demonstrated that the mean postoperative mortality was 7.1% (20). An earlier study revealed that the 30-day in-hospital mortality rate of pneumonectomy was 4.2%, the complication rate was 31.3%, and 5-year OS was 23.1% (21). Postpneumonectomy pulmonary edema and bronchopleural fistula are the most dangerous and potentially fatal complications after pneumonectomy, accounting for the majority of morbidity and mortality (22, 23). The high mortality and complication rates bring challenges to surgeons. With the development of minimally invasive techniques, pneumonectomy could be equally effective and less traumatic (24). Therefore, pneumonectomy should be considered a chance for the N2 patients rather than a calamity (25).

The development of new drugs or strategies of immunotherapy, target therapy, and chemotherapy, as well as the new techniques of radiotherapy like stereotactic body radiation therapy together led to remarkable progress in the survival of NSCLC patients in the past two decades (26); thus, we included year of diagnosis in this research to adjust these potential confounders in the multivariable analyses, and the adjusted HRs of pneumonectomy vs. no surgery for OS or CSS were all <1; moreover, the HRs in subgroups of year of diagnosis were consistent with the main results, meaning the development of therapies in these years did not weaken the survival benefit brought by pneumonectomy. Marital status was also verified to be independently associated with survival outcome in NSCLC, which had been reported in many studies (27–29). In our study, the adjusted-HR (95% CI) for married vs. single [OS: 0.931 (0.894–0.970) with *p* < 0.001 (**Table 2**), CSS: 0.943 (0.903–0.984) with *p* = 0.007 (**Table 3**)] suggested that married people had a better survival outcome, and this may be attributed to stronger mental or economic support from their spouses.

The limitations of this study must be noted. First, although we studied OS and CSS, the SEER database does not contain disease-free survival or short-term outcomes of pneumonectomy, including postoperative complications, life quality, and in-hospital mortality. Second, this study is a retrospective study with inevitable selection bias even with PSM conducted. Last, similar to other studies based on the SEER database, much vital information for NSCLC was absent from the database, such as information on sequence between chemotherapy and surgery or radiotherapy, cigarette usage, cardiac or pulmonary function, laboratory testing, imagological examination, thoracoscope usage, targeted therapy, or immunotherapy regimen, which might affect the final results.

In conclusion, for T1–4N2M0 NSCLC patients, pneumonectomy is associated with better long-term survival. Comprehensive evaluation and multidisciplinary assessment should be conducted for potential candidates for pneumonectomy.

## Data Availability Statement

The dataset supporting the conclusions of this article is available in the SEER*Stat software (version 8.3.9; https://seer.cancer.gov/resources/).

## Ethics Statement

Ethical review and approval were not required for the study on human participants in accordance with the local legislation and institutional requirements. Written informed consent for participation was not required for this study in accordance with the national legislation and the institutional requirements.

## Author Contributions

Conception/Design: SW, XL, and ML. Collection and/or assembly of data: JW, DF and ML. Data analysis and interpretation: SW, QW, and WZ. Manuscript writing: SW, QW, and ML. Final approval of manuscript: All authors. Funding support: XL. All authors contributed to the article and approved the submitted version.

## Funding

This study was funded by Program of Shanghai Academic Research Leader (No. 21XD1402800) and Shanghai “Rising Stars of Medical Talent” Youth Development Program: Outstanding Youth Medical Talents.

## Conflict of Interest

The authors declare that the research was conducted in the absence of any commercial or financial relationships that could be construed as a potential conflict of interest.

## Publisher’s Note

All claims expressed in this article are solely those of the authors and do not necessarily represent those of their affiliated organizations, or those of the publisher, the editors and the reviewers. Any product that may be evaluated in this article, or claim that may be made by its manufacturer, is not guaranteed or endorsed by the publisher.

## Abbreviations

AJCC, American Joint Committee on Cancer; CI, confidence interval; CRTS, neoadjuvant chemoradiotherapy or chemotherapy plus surgery; CSS, cancer-specific survival; dCRT, definitive chemoradiotherapy; HR, hazard ratio; ICD-O-3, International Classification of Diseases for Oncology; IQR, interquartile range; NSCLC, non-small cell lung cancer; OS, overall survival; PSM, propensity score matching; SEER, Surveillance, Epidemiology, and End Results.
