# Supplementary material for: Role of Pneumonectomy in T1–4N2M0 Non-Small Cell Lung Cancer: A Propensity Score Matching Analysis
Source: Front Oncol. 2022 Jun 20;12:880515. doi: 10.3389/fonc.2022.880515 (PMC9251381; doi:10.3389/fonc.2022.880515)
Supplement: Supplementary file 1 [file Table_1.docx]

Supplementary Material

**TABLE S1 Selection procedure of study cohort**

| Step | Criteria | Number excluded | Number remained |
| --- | --- | --- | --- |
| 1 | Patients with T1-4N2M0 NSCLC between 2004 and 2015 | - | 49,062 |
| 2 | Include if identified as only one primary tumor | 14,209 | 34,853 |
| 3 | Include if aged ≥18 years | 3 | 34,850 |
| 4 | Include if treated with no surgery or pneumonectomy | 7,111 | 27,739 |
| 5 | Exclude if diagnosed with autopsy/death certificate only or diagnosed without pathologically confirmed | 279 | 27,460 |
| 6 | Exclude if missing information about variables required by our study: 36 with unknown race, 131 with unknown laterality, 289 with unknown whether radiotherapy, 206 unknown cause of death was performed. | 662 | 26,798 |

NSCLC, non-small cell lung cancer.

**TABLE S2** Baseline characteristics of stage T1-4N2M0 NSCLC patients underwent chemoradiotherapy or single pneumonectomy

| **Variables** | **Before PSM** | | |  | **After PSM** | | |
| --- | --- | --- | --- | --- | --- | --- | --- |
|  | **Chemoradiotherapy n=13,803** | **Pneumonectomy n=222** | **P** |  | **Chemoradiotherapy n=202** | **Pneumonectomy n=202** | **P** |
| **Year of diagnosis** |  |  | <0.001 |  |  |  | 0.677 |
| 2004-2009 | 6191 (44.9%) | 147 (66.2%) |  |  | 133 (65.8%) | 129 (63.9%) |  |
| 2010-2015 | 7612 (55.1%) | 75 (33.8%) |  |  | 69 (34.2%) | 73 (36.1%) |  |
| **Age** |  |  | 0.101 |  |  |  | 0.803 |
| <64 years old | 5648 (40.9%) | 104 (46.8%) |  |  | 95 (47.0%) | 94 (46.5%) |  |
| 64-76years old | 6159 (44.6%) | 95 (42.8%) |  |  | 88 (43.6%) | 85 (42.1%) |  |
| >76 years old | 1996 (14.5%) | 23 (10.4%) |  |  | 19 (9.4%) | 23 (11.4%) |  |
| **Gender** |  |  | 0.004 |  |  |  | 0.67 |
| Male | 8077 (58.5%) | 151 (68.0%) |  |  | 135 (66.8%) | 139 (68.8%) |  |
| Female | 5726 (41.5%) | 71 (32.0%) |  |  | 67 (33.2%) | 63 (31.2%) |  |
| **Race** |  |  | 0.21 |  |  |  | 0.7 |
| White | 11045 (80.0%) | 182 (82.0%) |  |  | 173 (85.6%) | 168 (83.2%) |  |
| Black | 1989 (14.4%) | 24 (10.8%) |  |  | 17 (8.4%) | 22 (10.9%) |  |
| Other | 769 (5.6%) | 16 (7.2%) |  |  | 12 (5.9%) | 12 (5.9%) |  |
| **Marital status** |  |  | 0.578 |  |  |  | 0.78 |
| Single | 1900 (13.8%) | 26 (11.7%) |  |  | 31 (15.3%) | 24 (11.9%) |  |
| Married | 7465 (54.1%) | 119 (53.6%) |  |  | 105 (52.0%) | 109 (54.0%) |  |
| Seperated/Divorced/Widowed | 3950 (28.6%) | 71 (32.0%) |  |  | 61 (30.2%) | 63 (31.2%) |  |
| Unknown | 488 (3.5%) | 6 (2.7%) |  |  | 5 (2.5%) | 6 (3.0%) |  |
| **Laterality** |  |  | <0.001 |  |  |  | 0.764 |
| Right | 8852 (64.1%) | 93 (41.9%) |  |  | 91 (45.0%) | 88 (43.6%) |  |
| Left | 4951 (35.9%) | 129 (58.1%) |  |  | 111 (55.0%) | 114 (56.4%) |  |
| **Primary site** |  |  | <0.001 |  |  |  | 0.956 |
| Main bronchus | 942 (6.8%) | 19 (8.6%) |  |  | 19 (9.4%) | 18 (8.9%) |  |
| Upper lobe | 8590 (62.2%) | 106 (47.7%) |  |  | 110 (54.5%) | 105 (52.0%) |  |
| Middle lobe | 554 (4.0%) | 8 (3.6%) |  |  | 6 (3.0%) | 8 (4.0%) |  |
| Lower lobe | 3000 (21.7%) | 58 (26.1%) |  |  | 56 (27.7%) | 56 (27.7%) |  |
| Overlapping lesion of lung | 128 (0.9%) | 25 (11.3%) |  |  | 7 (3.5%) | 9 (4.5%) |  |
| Unknown | 589 (4.3%) | 6 (2.7%) |  |  | 4 (2.0%) | 6 (3.0%) |  |
| **Histologic type** |  |  | 0.575 |  |  |  | 0.344 |
| Adenocarcinoma | 4688 (34.0%) | 72 (32.4%) |  |  | 70 (34.7%) | 61 (30.2%) |  |
| Squamous cell | 5803 (42.0%) | 101 (45.5%) |  |  | 97 (48.0%) | 95 (47.0%) |  |
| Other | 3312 (24.0%) | 49 (22.1%) |  |  | 35 (17.3%) | 46 (22.8%) |  |
| **Differentiation** |  |  | <0.001 |  |  |  | 0.934 |
| Grade I | 283 (2.1%) | 11 (5.0%) |  |  | 13 (6.4%) | 9 (4.5%) |  |
| Grade II | 2105 (15.3%) | 66 (29.7%) |  |  | 58 (28.7%) | 61 (30.2%) |  |
| Grade III | 4419 (32.0%) | 122 (55.0%) |  |  | 110 (54.5%) | 110 (54.5%) |  |
| Grade IV | 249 (1.8%) | 4 (1.8%) |  |  | 3 (1.5%) | 3 (1.5%) |  |
| Unknown | 6747 (48.9%) | 19 (8.6%) |  |  | 18 (8.9%) | 19 (9.4%) |  |
| **T** |  |  | <0.001 |  |  |  | 0.634 |
| T1 | 1912 (13.9%) | 15 (6.8%) |  |  | 10 (5.0%) | 15 (7.4%) |  |
| T2 | 3554 (25.7%) | 81 (36.5%) |  |  | 73 (36.1%) | 76 (37.6%) |  |
| T3 | 3195 (23.1%) | 54 (24.3%) |  |  | 53 (26.2%) | 45 (22.3%) |  |
| T4 | 5142 (37.3%) | 72 (32.4%) |  |  | 66 (32.7%) | 66 (32.7%) |  |
| **Regional nodes examined** |  |  | <0.001 |  |  |  | <0.001 |
| 0-6 | 10898 (79.0%) | 46 (20.7%) |  |  | 178 (88.1%) | 45 (22.3%) |  |
| 7-23 | 203 (1.5%) | 130 (58.6%) |  |  | 2 (1.0%) | 116 (57.4%) |  |
| >23 | 26 (0.2%) | 24 (10.8%) |  |  | 0 (0.0%) | 21 (10.4%) |  |
| Unknown | 2676 (19.4%) | 22 (9.9%) |  |  | 22 (10.9%) | 20 (9.9%) |  |
| **Regional nodes positive** |  |  | <0.001 |  |  |  | <0.001 |
| 0 | 362 (2.6%) | 10 (4.5%) |  |  | 5 (2.5%) | 10 (5.0%) |  |
| 1-5 | 1498 (10.9%) | 137 (61.7%) |  |  | 21 (10.4%) | 127 (62.9%) |  |
| >5 | 69 (0.5%) | 56 (25.2%) |  |  | 1 (0.5%) | 47 (23.3%) |  |
| Unknown | 11874 (86.0%) | 19 (8.6%) |  |  | 175 (86.6%) | 18 (8.9%) |  |
| **All-cause death** | 11243 (81.5%) | 183 (82.4%) | 0.71 |  | 184 (91.1%) | 165 (81.7%) | 0.006 |
| **Cancer-specific death** | 10132 (73.4%) | 148 (66.7%) | 0.024 |  | 160 (79.2%) | 134 (66.3%) | 0.004 |

Categorical variables are presented with number (percentage). NSCLC, non-small cell lung cancer; PSM, propensity score matching.

**TABLE S3** Cox regression analysis of the influence of pneumonectomy on OS in stage T1-4N2M0 NSCLC patients underwent chemoradiotherapy or single pneumonectomy

| **Variables** | **Before PSM** | | | |  | **After PSM** | | | |
| --- | --- | --- | --- | --- | --- | --- | --- | --- | --- |
|  | **Univariable analysis** | | **Multivariable analysis** | |  | **Univariable analysis** | | **Multivariable analysis** | |
|  | **HR (95%CI)** | **P** | **HR (95%CI)** | **P** |  | **HR (95%CI)** | **P** | **HR (95%CI)** | **P** |
| **Surgery** |  |  |  |  |  |  |  |  |  |
| No | 1 |  |  |  |  | 1 |  |  |  |
| Pneumonectomy | 1.016 (0.878-1.176) | 0.832 |  |  |  | 0.988 (0.799-1.222) | 0.913 |  |  |
| **Year of diagnosis** |  |  |  |  |  |  |  |  |  |
| 2004-2009 | 1 |  | 1 |  |  | 1 |  |  |  |
| 2010-2015 | 0.849 (0.818-0.881) | <0.001 | 0.851 (0.819-0.885) | <0.001 |  | 0.899 (0.716-1.129) | 0.36 |  |  |
| **Age** |  |  |  |  |  |  |  |  |  |
| <64 years old | 1 |  | 1 |  |  | 1 |  | 1 |  |
| 64-76years old | 1.132 (1.088-1.178) | <0.001 | 1.156 (1.110-1.204) | <0.001 |  | 1.096 (0.876-1.370) | 0.422 | 1.013 (0.798-1.285) | 0.917 |
| >76 years old | 1.286 (1.216-1.359) | <0.001 | 1.308 (1.235-1.384) | <0.001 |  | 1.548 (1.090-2.199) | 0.015 | 1.525 (1.060-2.194) | 0.023 |
| **Gender** |  |  |  |  |  |  |  |  |  |
| Male | 1 |  | 1 |  |  | 1 |  | 1 |  |
| Female | 0.819 (0.789-0.851) | <0.001 | 0.827 (0.795-0.860) | <0.001 |  | 0.738 (0.587-0.928) | 0.009 | 0.830 (0.648-1.062) | 0.138 |
| **Race** |  |  |  |  |  |  |  |  |  |
| White | 1 |  | 1 |  |  | 1 |  |  |  |
| Black | 0.881 (0.836-0.930) | <0.001 | 0.905 (0.857-0.956) | <0.001 |  | 0.843 (0.582-1.221) | 0.366 |  |  |
| Other | 0.861 (0.793-0.935) | <0.001 | 0.860 (0.792-0.934) | <0.001 |  | 1.349 (0.881-2.064) | 0.168 |  |  |
| **Marital status** |  |  |  |  |  |  |  |  |  |
| Single | 1 |  | 1 |  |  | 1 |  |  |  |
| Married | 1.049 (0.992-1.110) | 0.093 | 0.947 (0.893-1.004) | 0.066 |  | 0.920 (0.665-1.272) | 0.614 |  |  |
| Seperated/Divorced/Widowed | 1.069 (1.006-1.136) | 0.03 | 1.018 (0.956-1.084) | 0.583 |  | 1.045 (0.741-1.474) | 0.802 |  |  |
| Unknown | 1.001 (0.894-1.120) | 0.992 | 0.975 (0.870-1.092) | 0.663 |  | 0.856 (0.431-1.697) | 0.656 |  |  |
| **Laterality** |  |  |  |  |  |  |  |  |  |
| Right | 1 |  |  |  |  | 1 |  |  |  |
| Left | 0.997 (0.960-1.036) | 0.894 |  |  |  | 0.911 (0.737-1.126) | 0.39 |  |  |
| **Primary site** |  |  |  |  |  |  |  |  |  |
| Main bronchus | 1 |  | 1 |  |  | 1 |  | 1 |  |
| Upper lobe | 0.895 (0.832-0.963) | 0.003 | 0.930 (0.864-1.001) | 0.054 |  | 0.524 (0.361-0.760) | <0.001 | 0.447 (0.304-0.658) | <0.001 |
| Middle lobe | 1.029 (0.919-1.153) | 0.622 | 1.135 (1.012-1.272) | 0.031 |  | 0.708 (0.366-1.370) | 0.305 | 1.051 (0.530-2.088) | 0.886 |
| Lower lobe | 0.987 (0.912-1.069) | 0.752 | 1.046 (0.965-1.134) | 0.272 |  | 0.594 (0.401-0.880) | 0.009 | 0.530 (0.354-0.794) | 0.002 |
| Overlapping lesion of lung | 1.118 (0.930-1.345) | 0.234 | 1.071 (0.890-1.287) | 0.469 |  | 0.798 (0.434-1.468) | 0.468 | 0.841 (0.448-1.578) | 0.589 |
| Unknown | 1.020 (0.911-1.141) | 0.737 | 1.042 (0.931-1.167) | 0.475 |  | 0.288 (0.120-0.691) | 0.005 | 0.241 (0.098-0.593) | 0.002 |
| **Histologic type** |  |  |  |  |  |  |  |  |  |
| Adenocarcinoma | 1 |  | 1 |  |  | 1 |  | 1 |  |
| Squamous cell | 1.277 (1.223-1.333) | <0.001 | 1.161 (1.110-1.214) | <0.001 |  | 1.249 (0.984-1.585) | 0.067 | 1.049 (0.812-1.355) | 0.715 |
| Other | 1.223 (1.165-1.284) | <0.001 | 1.121 (1.065-1.179) | <0.001 |  | 0.800 (0.587-1.089) | 0.157 | 0.768 (0.548-1.075) | 0.124 |
| **Differentiation** |  |  |  |  |  |  |  |  |  |
| Grade I | 1 |  | 1 |  |  | 1 |  | 1 |  |
| Grade II | 1.145 (0.999-1.313) | 0.051 | 1.060 (0.924-1.216) | 0.406 |  | 2.405 (1.351-4.283) | 0.003 | 2.164 (1.192-3.927) | 0.011 |
| Grade III | 1.151 (1.009-1.314) | 0.037 | 1.078 (0.943-1.231) | 0.272 |  | 2.571 (1.465-4.513) | 0.001 | 2.434 (1.365-4.339) | 0.003 |
| Grade IV | 1.319 (1.097-1.586) | 0.003 | 1.195 (0.991-1.440) | 0.062 |  | 2.960 (1.052-8.326) | 0.04 | 2.569 (0.884-7.465) | 0.083 |
| Unknown | 1.069 (0.938-1.219) | 0.315 | 1.026 (0.899-1.171) | 0.701 |  | 1.963 (1.023-3.765) | 0.042 | 2.012 (1.030-3.933) | 0.041 |
| **T** |  |  |  |  |  |  |  |  |  |
| T1 | 1 |  | 1 |  |  | 1 |  | 1 |  |
| T2 | 1.197 (1.125-1.274) | <0.001 | 1.150 (1.080-1.225) | <0.001 |  | 1.798 (1.083-2.986) | 0.023 | 1.534 (0.911-2.583) | 0.107 |
| T3 | 1.265 (1.187-1.349) | <0.001 | 1.226 (1.149-1.308) | <0.001 |  | 2.089 (1.240-3.519) | 0.006 | 1.793 (1.050-3.060) | 0.032 |
| T4 | 1.407 (1.326-1.493) | <0.001 | 1.372 (1.291-1.458) | <0.001 |  | 2.491 (1.494-4.154) | <0.001 | 2.249 (1.316-3.844) | 0.003 |

OS, overall survival; NSCLC, non-small cell lung cancer; PSM, propensity score matching; HR, hazard ratio; CI, confidence interval.

**TABLE S4** Cox regression analysis of the influence of pneumonectomy on CSS in stage T1-4N2M0 NSCLC patients underwent chemoradiotherapy or single pneumonectomy

| **Variables** | **Before PSM** | | | |  | **After PSM** | | | |
| --- | --- | --- | --- | --- | --- | --- | --- | --- | --- |
|  | **Univariable analysis** | | **Multivariable analysis** | |  | **Univariable analysis** | | **Multivariable analysis** | |
|  | **HR (95%CI)** | **P** | **HR (95%CI)** | **P** |  | **HR (95%CI)** | **P** | **HR (95%CI)** | **P** |
| **Surgery** |  |  |  |  |  |  |  |  |  |
| No | 1 |  |  |  |  | 1 |  |  |  |
| Pneumonectomy | 0.934 (0.794-1.099) | 0.413 |  |  |  | 0.938 (0.744-1.182) | 0.586 |  |  |
| **Year of diagnosis** |  |  |  |  |  |  |  |  |  |
| 2004-2009 | 1 |  | 1 |  |  | 1 |  |  |  |
| 2010-2015 | 0.848 (0.815-0.881) | <0.001 | 0.851 (0.818-0.886) | <0.001 |  | 0.913 (0.714-1.168) | 0.469 |  |  |
| **Age** |  |  |  |  |  |  |  |  |  |
| <64 years old | 1 |  | 1 |  |  | 1 |  |  |  |
| 64-76years old | 1.080 (1.036-1.127) | <0.001 | 1.106 (1.060-1.153) | <0.001 |  | 1.047 (0.822-1.334) | 0.708 |  |  |
| >76 years old | 1.206 (1.137-1.279) | <0.001 | 1.235 (1.164-1.311) | <0.001 |  | 1.322 (0.886-1.974) | 0.172 |  |  |
| **Gender** |  |  |  |  |  |  |  |  |  |
| Male | 1 |  | 1 |  |  | 1 |  | 1 |  |
| Female | 0.823 (0.791-0.856) | <0.001 | 0.846 (0.812-0.880) | <0.001 |  | 0.727 (0.566-0.934) | 0.013 | 0.792 (0.612-1.025) | 0.077 |
| **Race** |  |  |  |  |  |  |  |  |  |
| White | 1 |  | 1 |  |  | 1 |  |  |  |
| Black | 0.885 (0.837-0.936) | <0.001 | 0.907 (0.858-0.960) | <0.001 |  | 0.861 (0.578-1.281) | 0.46 |  |  |
| Other | 0.893 (0.820-0.973) | 0.01 | 0.888 (0.815-0.967) | 0.006 |  | 1.170 (0.715-1.915) | 0.531 |  |  |
| **Marital status** |  |  |  |  |  |  |  |  |  |
| Single | 1 |  |  |  |  | 1 |  |  |  |
| Married | 1.040 (0.980-1.103) | 0.194 |  |  |  | 0.784 (0.557-1.103) | 0.162 |  |  |
| Seperated/Divorced/Widowed | 1.046 (0.981-1.115) | 0.168 |  |  |  | 0.973 (0.679-1.395) | 0.881 |  |  |
| Unknown | 0.968 (0.859-1.091) | 0.597 |  |  |  | 0.922 (0.463-1.836) | 0.818 |  |  |
| **Laterality** |  |  |  |  |  |  |  |  |  |
| Right | 1 |  |  |  |  | 1 |  |  |  |
| Left | 0.988 (0.949-1.028) | 0.545 |  |  |  | 0.847 (0.673-1.065) | 0.156 |  |  |
| **Primary site** |  |  |  |  |  |  |  |  |  |
| Main bronchus | 1 |  | 1 |  |  | 1 |  | 1 |  |
| Upper lobe | 0.889 (0.823-0.960) | 0.003 | 0.927 (0.858-1.002) | 0.056 |  | 0.522 (0.349-0.780) | 0.002 | 0.472 (0.312-0.714) | <0.001 |
| Middle lobe | 1.013 (0.898-1.141) | 0.837 | 1.126 (0.998-1.270) | 0.053 |  | 0.680 (0.331-1.399) | 0.295 | 1.043 (0.493-2.203) | 0.913 |
| Lower lobe | 0.975 (0.897-1.061) | 0.559 | 1.044 (0.960-1.136) | 0.316 |  | 0.572 (0.373-0.877) | 0.01 | 0.562 (0.364-0.867) | 0.009 |
| Overlapping lesion of lung | 1.080 (0.887-1.315) | 0.444 | 1.033 (0.848-1.258) | 0.75 |  | 0.878 (0.463-1.666) | 0.691 | 0.962 (0.496-1.869) | 0.91 |
| Unknown | 1.010 (0.897-1.138) | 0.863 | 1.026 (0.911-1.155) | 0.677 |  | 0.226 (0.079-0.648) | 0.006 |  |  |
| **Histologic type** |  |  |  |  |  |  |  |  |  |
| Adenocarcinoma | 1 |  | 1 |  |  | 1 |  |  |  |
| Squamous cell | 1.237 (1.182-1.294) | <0.001 | 1.122 (1.071-1.176) | <0.001 |  | 1.193 (0.921-1.545) | 0.181 |  |  |
| Other | 1.214 (1.153-1.277) | <0.001 | 1.107 (1.049-1.168) | <0.001 |  | 0.794 (0.569-1.109) | 0.176 |  |  |
| **Differentiation** |  |  |  |  |  |  |  |  |  |
| Grade I | 1 |  | 1 |  |  | 1 |  | 1 |  |
| Grade II | 1.161 (1.003-1.342) | 0.045 | 1.080 (0.933-1.249) | 0.304 |  | 2.276 (1.212-4.271) | 0.01 | 2.341 (1.233-4.445) | 0.009 |
| Grade III | 1.185 (1.029-1.364) | 0.018 | 1.106 (0.960-1.274) | 0.164 |  | 2.679 (1.454-4.937) | 0.002 | 2.654 (1.425-4.943) | 0.002 |
| Grade IV | 1.333 (1.096-1.622) | 0.004 | 1.198 (0.982-1.462) | 0.074 |  | 3.751 (1.299-10.831 | 0.015 | 3.125 (1.065-9.175) | 0.038 |
| Unknown | 1.098 (0.955-1.263) | 0.19 | 1.057 (0.919-1.217) | 0.437 |  | 2.038 (1.006-4.127) | 0.048 | 2.018 (0.978-4.163) | 0.058 |
| **T** |  |  |  |  |  |  |  |  |  |
| T1 | 1 |  | 1 |  |  | 1 |  | 1 |  |
| T2 | 1.247 (1.166-1.334) | <0.001 | 1.202 (1.123-1.287) | <0.001 |  | 1.850 (1.059-3.233) | 0.031 | 1.654 (0.937-2.922) | 0.083 |
| T3 | 1.348 (1.258-1.443) | <0.001 | 1.310 (1.222-1.405) | <0.001 |  | 2.012 (1.131-3.580) | 0.017 | 1.875 (1.046-3.362) | 0.035 |
| T4 | 1.530 (1.436-1.631) | <0.001 | 1.494 (1.400-1.595) | <0.001 |  | 2.678 (1.529-4.693) | <0.001 | 2.631 (1.474-4.696) | 0.001 |

CSS, overall survival; NSCLC, non-small cell lung cancer; PSM, propensity score matching; HR, hazard ratio; CI, confidence interval.

**FIGURE LEGENDS**

**FIGURE S1** Kaplan-Meier estimates of OS and CSS for stage T1-4N2M0 NSCLC patients comparing chemoradiotherapy with single pneumonectomy: OS before PSM (A), OS after PSM (B), CSS before PSM (C), and CSS after PSM (D). OS, overall survival; CSS, cancer-specific survival; NSCLC, non-small cell lung cancer, PSM, propensity score matching.
