# Supplementary figures and images for: Role of Pneumonectomy in T1–4N2M0 Non-Small Cell Lung Cancer: A Propensity Score Matching Analysis
Source: Front Oncol. 2022 Jun 20;12:880515. doi: 10.3389/fonc.2022.880515 (PMC9251381; doi:10.3389/fonc.2022.880515)

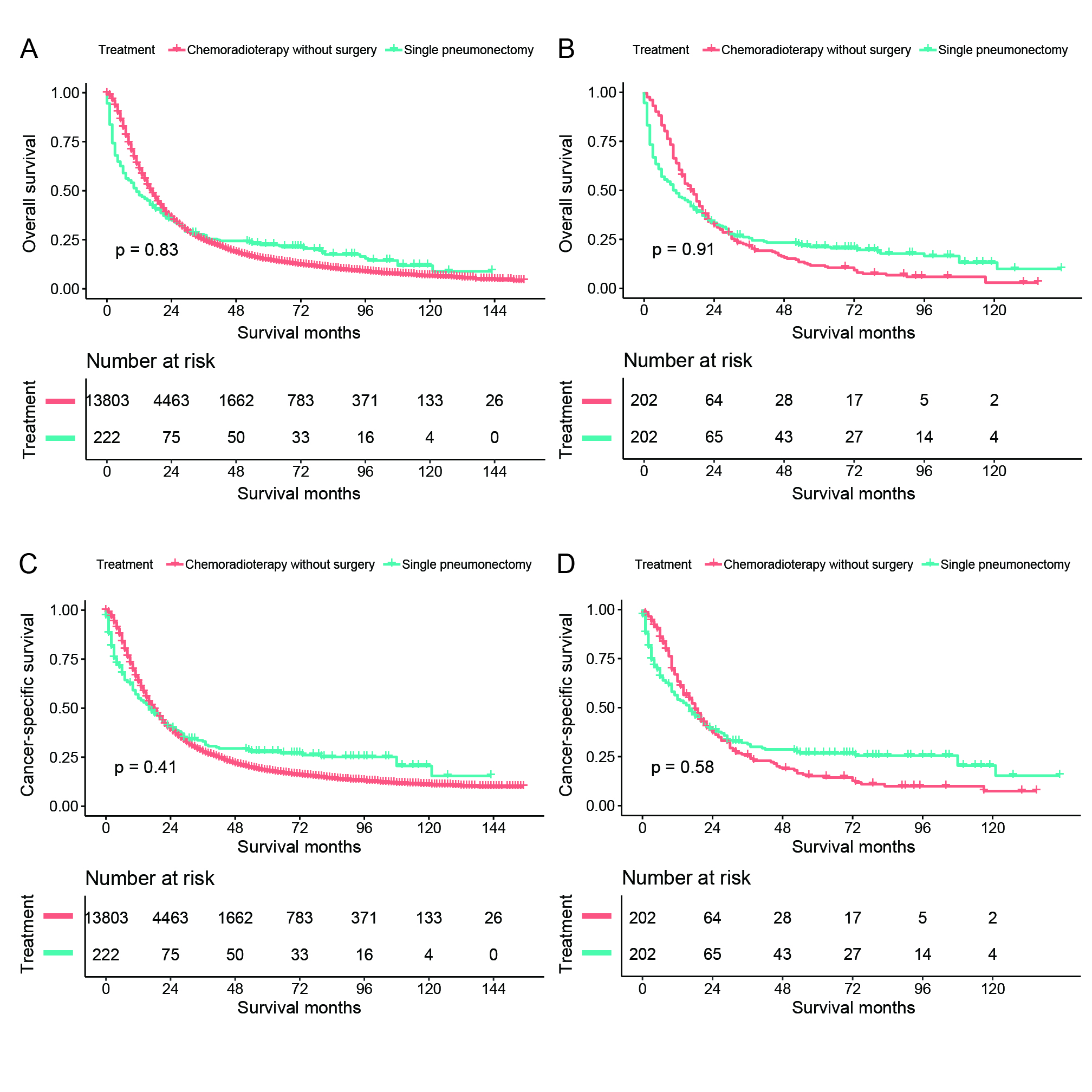

Supplement: Supplementary file 2 [file Image_1.jpeg]
